# NSAID Use Attenuates the Protective Effect of Physical Activity on Chronic Low Back Pain: A Cross-Sectional Analysis of NHANES 2009–2010

**DOI:** 10.3390/biomedicines14051165

**Published:** 2026-05-21

**Authors:** William Sosa, Lucas Camargo, Felipe Fregni

**Affiliations:** Spaulding Neuromodulation Center and Center for Clinical Research Learning, Spaulding Rehabilitation Hospital, Harvard Medical School, Boston, MA 02115, USA; wsosa1@mgb.org (W.S.); lcamargo@mgh.harvard.edu (L.C.)

**Keywords:** chronic low back pain, physical activity, NSAIDs, effect modification, NHANES

## Abstract

**Background**: Chronic low back pain (CLBP) is a leading cause of disability worldwide, with exercise endorsed as first-line treatment and non-steroidal anti-inflammatory drugs (NSAIDs) among the most used pharmacologic options. These interventions are frequently combined in clinical practice, yet their synergistic effects remain unclear. To evaluate whether NSAID use modifies the association between physical activity (PA) and CLBP using nationally representative data from NHANES 2009–2010. **Methods**: We analyzed 988 adults aged ≥20 years with complete data on chronic low back pain, physical activity, medication use, and modeled covariates. **Results**: Among participants not using NSAIDs, moderate recreational physical activity was associated with lower odds of CLBP (adjusted OR = 0.47, 95% CI 0.25–0.91; *p* = 0.029). Active transport showed a similar direction but was not statistically significant (OR = 0.38, 95% CI 0.13–1.12; *p* = 0.074). In interaction models, active transport x aspirin was associated with higher odds of CLBP (OR = 2.24, 95% CI 1.02–4.90; *p* = 0.044), and moderate recreational PA x any NSAID use was also associated with higher odds of CLBP (OR = 2.26, 95% CI 1.01–5.06; *p* = 0.047). Subgroup analyses were exploratory and heterogeneous, including a significant potential protective interaction (OR ≈ 0.19, 95% CI 0.06–0.69; *p* = 0.015). **Conclusions**: In a nationally representative sample, NSAID use appeared to modify the association between physical activity and chronic low back pain. These findings are exploratory and hypothesis-generating. Therefore, longitudinal studies are needed to clarify the temporal and causal relationships and the potential influence of NSAIDs.

## 1. Introduction

Chronic low back pain (CLBP) is a leading cause of disability and medical costs worldwide. Recent analyses from the Global Burden of Disease 2021 Study estimate that low back pain affected approximately 629 million individuals globally in 2021, remaining the leading cause of years lived with disability across all age groups [1]. Among older adults, CLBP is particularly prevalent, with systematic reviews reporting associations with obesity, depression, anxiety, multiple comorbidities, and functional limitations [2].

Clinical guidelines consistently emphasize non-pharmacological management, with exercise therapy recommended as first-line treatment across multiple international guidelines [3]. Structured exercise programs produce clinically meaningful reductions in pain and functional limitation across a broad range of modalities: a 2021 network meta-analysis of 217 randomized controlled trials found that exercise consistently outperformed minimal care with moderate to large effect sizes [4], and a 2023 meta-analysis confirmed significant improvements in pain and function across 20 distinct exercise regimens, regardless of exercise type [5]. Among older adults specifically, exercise has been shown to improve pain intensity, mobility, and quality of life [6]. Despite this evidence base, NSAIDs remain among the most used pharmacologic treatments for musculoskeletal pain and are frequently combined with exercise in clinical practice [7].

The biological pathways through which exercise protects against chronic pain are multiple and only partially understood. Beyond its well-recognized mechanical and psychological effects, physical activity activates a tightly regulated neuroimmune response that appears necessary for pain resolution. Prostaglandin and eicosanoid signaling, inflammasome activation, and cytokine production each contribute to remodeling of the nociceptive system [8,9,10,11], while repeated exercise bouts drive neuroplastic changes in descending pain-inhibitory circuits, motor cortical representations, and affective pain-processing networks. Because NSAIDs inhibit COX enzymes and broadly modify eicosanoid and cytokine profiles, they have the potential to interfere with several of these adaptive pathways simultaneously, not only through anti-inflammatory action but through downstream effects on central sensitization, glial reactivity, and sensorimotor feedback.

Importantly, exercise and NSAIDs may each reduce pain through partially distinct mechanisms. Exercise can protect against chronic pain by improving tissue tolerance, strengthening muscles, increasing proprioceptive input, reducing fear-avoidance behavior, and enhancing descending pain inhibition [12,13]. Exercise also induces transient cytokine, prostaglandin, and immune responses that contribute to tissue remodeling and adaptive neuroimmune recalibration [14]. However, these pathways may overlap in clinically important ways, because prostaglandin and inflammatory signals are also involved in exercise-induced adaptation [15,16]. Thus, when NSAIDs are used concurrently, especially repeatedly or chronically, they may theoretically blunt adaptive inflammatory and sensory signals, reducing or even reversing the protective association between physical activity and CLBP in susceptible subgroups. 

Evidence for this interference is emerging from multiple lines of research. Parisien et al. [8] demonstrated that neutrophil-driven inflammation after acute back injury promotes pain resolution, whereas NSAID use delays recovery, suggesting that blunting natural inflammatory signals may dysregulate endogenous pain-healing mechanisms. Conversely, Trappe et al. [17] found that older adults using ibuprofen or acetaminophen during resistance training still achieved normal muscle hypertrophy and strength gains, highlighting that NSAID effects on exercise adaptation are context-dependent and not uniformly suppressive. Conditioned pain modulation—a measure of descending inhibitory efficiency that exercise is known to enhance—has also been shown to be disrupted by analgesic medication use [18,19], suggesting that pharmacological interference with exercise-induced analgesia extends beyond peripheral inflammation to central pain regulatory mechanisms.

Despite the clinical ubiquity of concurrent NSAID and exercise use in CLBP management, whether NSAIDs modify the protective association between physical activity and CLBP at the population level remains unexamined. We therefore analyzed NHANES 2009–2010 data to investigate this question using a nationally representative sample. Despite the limitations of this dataset, this cycle uniquely includes concurrent measures of physical activity, NSAID use, and chronic low back pain, allowing examination of an interaction that has not been previously studied at the population level. We hypothesized that NSAID use would attenuate or reverse the protective association between physical activity and CLBP, and further explored whether this effect modification varies across subpopulations known to differ in neuroimmune status, pain vulnerability, and inflammatory baseline, including older adults, individuals with depression or obesity, and those with hypertension.

## 2. Materials and Methods

### 2.1. Data Source and Study Design

This study is a cross-section, survey-weighted, observational secondary analysis of data from the 2009–2010 cycle of the National Health and Nutrition Examination Survey (NHANES), a cross-sectional survey designed to provide nationally representative health information for the non-institutionalized U.S. population. NHANES uses a complex, multistage probability sampling design with stratification, clustering, and oversampling of selected subgroups. Publicly available, de-identified data files were obtained from the Centers for Disease Control and Prevention. We tested if NSAID use modifies the association between physical activity and chronic low back pain. Subgroup interaction analyses were considered exploratory. Given the cross-sectional design, temporal relationships between exposure and outcome cannot be established.

### 2.2. Study Sample

Adults aged 20 years or older were eligible for inclusion. The analytic sample was restricted to participants with complete data on chronic low back pain (CLBP), physical activity, NSAID use, and covariates required for the regression models. The analytic sample was derived by merging NHANES 2009–2010 datasets for physical activity (PAQ), analgesic use (ARQ), demographics (DEMO), body composition (BMX), smoking (SMQ), depression (DPQ), and blood pressure (BPQ) using the participant identifier (SEQN). Participants were first restricted to those with valid physical activity data. Chronic low back pain (CLBP) was then defined using NHANES questionnaire items capturing pain duration and chronicity, and individuals with missing CLBP status were excluded. In addition, complete data on all NSAID exposure variables (ibuprofen, naproxen, indomethacin, COX-2 inhibitors, and aspirin) were required to ensure consistent exposure classification. This complete-case restriction resulted in a final analytic sample of 988 participants. While this approach ensures internal consistency of regression models, it may limit generalizability.

### 2.3. Outcome Definition

The outcome was chronic low back pain (CLBP), coded as a binary variable. CLBP was defined using the study’s prespecified NHANES back-pain items corresponding to chronic back pain duration greater than 3 months. This definition is based on self-reported data and may be subject to misclassification. Participants meeting this definition were coded as having CLBP; all others were coded as not having CLBP.

### 2.4. Physical Activity Variables

Physical activity (PA) exposures were derived from NHANES Physical Activity Questionnaire items and categorized into the following domains: active transport, moderate recreational activity, vigorous recreational activity, moderate work activity, and vigorous work activity. Each PA domain was coded dichotomously as yes/no. Active transport was defined as walking or bicycling for ≥10 min per day, consistent with NHANES PAQ coding. Additional derived PA variables were explored during model development, but the primary analyses focused on these prespecified activity domains.

### 2.5. NSAID Exposure

NSAID exposure was defined using self-reported medication variables. Individual NSAID categories included ibuprofen, naproxen, indomethacin, COX-2 inhibitors, and aspirin. A composite “any NSAID use” variable was defined as use of any of these medications.

### 2.6. Covariates

Prespecified covariates included sex, age group (≥60 years), smoking status, obesity, depression, hypertension, education, and marital/partner status. Obesity was defined as body mass index (BMI) ≥ 30 kg/m^2^. Depression was defined as a Patient Health Questionnaire-9 (PHQ-9) score ≥ 10. Hypertension was based on self-reported history. Education and marital/partner status were coded using NHANES demographic variables.

### 2.7. Statistical Analysis

Descriptive results are presented as unweighted counts and survey-weighted prevalence estimates. Table 1 reports unweighted frequencies by CLBP status and weighted percentages for the analytic sample. Regression analyses were conducted using survey-weighted logistic regression to account for the NHANES complex sampling design. Interview weights (WTINT2YR), strata (SDMVSTRA), and primary sampling units (SDMVPSU) were applied in all models. Because the outcome, physical activity variables, and medication exposures were questionnaire-based, interview weights were used throughout.

Effect modification by NSAID use was assessed by including interaction terms between each physical activity variable and each NSAID exposure. To facilitate interpretation, interaction models were used to estimate effect modification, with the coefficient for physical activity representing the adjusted association among non-NSAID users (reference stratum). Models restricted to non-NSAID users were additionally fitted as confirmatory analyses to ensure consistency of the baseline effect across model specifications.

A complete-case analytic approach was used, restricting the dataset to participants with non-missing values for all variables included in the final models. Subgroup analyses were conducted for prespecified strata including older adults, women, smoking status, obesity, depression, hypertension, and marital/partner status. Given the exploratory nature of subgroup interaction analyses and the potential for sparse cells in some strata, large subgroup-specific ORs were interpreted cautiously. All analyses were conducted in R version 4.5.0 using the survey, haven, tidyverse, and openxlsx packages.

### 2.8. Ethical Considerations

Because this study used publicly available, de-identified NHANES data, it was considered exempt from institutional review board approval in accordance with federal guidelines for secondary data analysis.

## 3. Results

### 3.1. Cohort Description

The analytic sample included 988 adults age 20 years or older with complete data on chronic low back pain, physical activity, and NSAID medication use. Table 1 presents weighted counts and survey-weighted prevalence estimates for the analytic sample. Weighted CLBP prevalence was 80.4% (±2.0 SE; 95% CI 76–84). Any NSAID use was 85.5%, ibuprofen 78.8%, naproxen 53.1%, and aspirin 33.0%. For physical activity, 21.4% walked or biked for transport ≥ 10 min/day, 58.2% performed moderate recreational PA, 17.9% vigorous recreational PA, 48.3% moderate work PA, and 26.3% vigorous work PA.

Unweighted frequencies and proportions by CLBP status are presented in Appendix A, Table A1. The cohort was 51.7% female and 55.8% current or former smokers. About 21% of participants were aged ≥60 years, and approximately 20% screened positive for depression. Nearly half of the sample (∼48%) met criteria for obesity (BMI ≥ 30). Most participants (85%) reported regular NSAID use and 81% met criteria for CLBP. Ibuprofen and naproxen were the most frequently reported individual NSAIDs, reported by approximately 77–79% and 51–53% of participants, respectively. Regarding physical activity, 24% of respondents walked or biked for transport at least 10 min per day; 38% engaged in moderate recreational activity and 15% in vigorous recreational activity. The high prevalence of chronic low back pain in the analytic sample reflects complete-case selection based on outcome, exposure, and covariate availability and should not be interpreted as population prevalence.

### 3.2. Main Effects and Interactions

**Figure 1:**
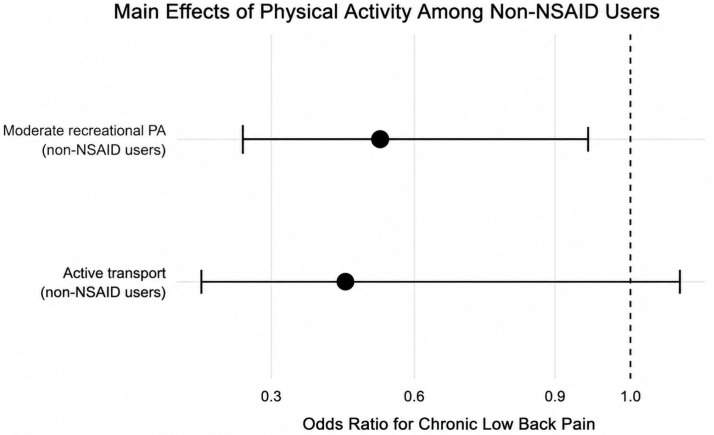
Survey-weighted odds ratios for chronic low back pain among non-NSAID users. Moderate recreational physical activity was associated with lower odds of chronic low back pain, while active transport showed a non-significant protective trend. Error bars (solid line) represent 95% confidence intervals, and the dashed vertical line indicates the null interaction value, OR = 1.

Among participants not using NSAIDs, models demonstrated that moderate recreational physical activity was associated with lower odds of CLBP (OR = 0.47, 95% CI 0.25–0.91; *p* = 0.029). Active transport showed a similar direction but did not reach statistical significance (OR = 0.38, 95% CI 0.13–1.12; *p* = 0.074), as displayed in Figure 1.

### 3.3. Effect Modification by NSAID Use

**Figure 2:**
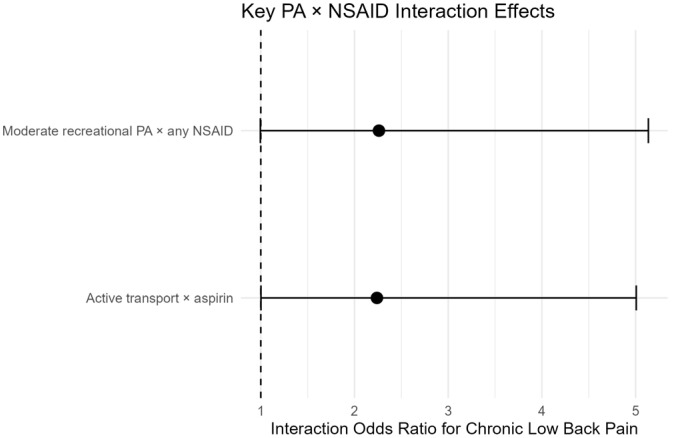
Survey-weighted interaction odds ratios for chronic low back pain. Active transport × aspirin and moderate recreational physical activity × any NSAID use were associated with increased odds of chronic low back pain, indicating possible attenuation or reversal of the protective effect of physical activity. Error bars (solid line) represent 95% confidence intervals, and the dashed vertical line indicates the null interaction value, OR = 1.

Across multiple domains, NSAID use was associated with attenuation or modification of exercise’s protective association between exercise and CLBP. Active transport × aspirin interaction yielded (OR 2.24, 95% CI 1.02–4.90; *p* = 0.044), and moderate recreational PA × any NSAID (OR 2.26, 1.01–5.06; *p* = 0.047), as displayed in Figure 2. Subgroup analyses showed marked heterogeneity, with a protective interaction observed among smokers (OR 0.19, 95% CI 0.06–0.69; *p* = 0.015). Many subgroup-specific estimates were imprecise with wide confidence intervals because of the exploratory nature of the analysis. Expanded subgroup analyses and corresponding plots are provided in Appendix B.

## 4. Discussion

Physical activity exerts its protective effects against chronic pain through multiple, partially overlapping biological pathways. At the neurological level, repeated exercise promote central sensitization reversal through enhanced descending inhibitory control, upregulation of endogenous opioid and endocannabinoid tone, and cortical reorganization that reduces pain amplification [13,20]. Evidence from TMS-based studies demonstrates that exercise interventions significantly improve corticospinal excitability in pain populations (standardized mean difference = 2.06), suggesting that neuroplastic remodeling is a primary driver of exercise-induced analgesia [21]. Further supporting this view, motor task engagement has been shown to reduce pain perception through mechanisms that are dissociable from motor cortex excitability changes alone, implicating broader networks beyond primary motor cortex [22]. At the musculoskeletal level, mechanical loading drives tissue remodeling, improved proprioception, and reduced peripheral sensitization [23,24]. At the psychosocial level, exercise attenuates fear-avoidance behavior, improves self-efficacy, and reduces catastrophizing, all independent predictors of chronicity in low back pain [25]. Given these converging mechanisms through which exercise modulates pain, we discuss how NSAIDs may be associated with disruption of one or a combination of these pathways.

Our results align with and extend prior observational and interventional evidence on the interplay between physical activity, analgesics, and CLBP outcomes. A prospective cohort study found that engagement in moderate-vigorous physical activity was associated with reduced frequency of analgesic use for LBP, while higher physical workload and sedentary time were linked to greater analgesic reliance and activity limitation [26]. In a head-to-head prospective study of patients with chronic nonspecific low back pain, celecoxib monotherapy and home-based stretching/strengthening exercises produced comparable reductions in pain intensity after 3 months; however, only the exercise group showed statistically significant improvements in disability and select domains of quality of life [27]. Additionally, a benefit–harm trade-off study reported that people with chronic LBP consider an approximately 20–30% additional reduction in pain intensity (beyond natural history) as the smallest worthwhile effect for either NSAIDs or individualized exercise [28]. Biologically, several mechanisms could plausibly contribute to the observed pattern of effect modification, although our data cannot directly test these pathways.

Physical activity may have protective effects against chronic pain, in part through engagement of the motor cortex and descending pain-inhibitory circuits. Evidence from TMS-based studies confirms that exercise significantly improves corticospinal excitability in pain populations [21]. Critically, individuals with greater motor cortical plasticity reserve appear better protected against chronic pain development: complex motor imagery ability, a surrogate for intact motor cortical engagement, was inversely associated with phantom limb pain magnitude, suggesting that the capacity to flexibly recruit and remodel motor representations is itself analgesic [29,30]. Building on this framework, we propose that chronic NSAID use may be associated with the reduction in this protective pathway, not only through anti-inflammatory action, but also through possible disruption of the afferent sensory signals that normally drive motor cortex adaptation. Somatosensory input is not merely a byproduct of movement, it is an active driver of cortical plasticity and pain modulation. Volz et al. [22] demonstrated that somatosensory tasks modulate pain thresholds in a hand-specific manner and alter cortical excitability via subcortical and spinal circuits, independently of motor cortical mechanisms. Critically, when afferent input is reduced or distorted, this bottom-up drive to the pain modulatory system is weakened. NSAIDs, by blunting nociceptive and inflammatory signaling at the periphery, may effectively reduce the intensity of the afferent signaling that accompanies movement, depriving the motor cortex and its downstream inhibitory circuits of the very input needed to sustain neuroplastic adaptation [31,32,33]. Over time, this could produce a gradual reduction in descending inhibitory tone, leaving the pain system progressively less regulated and more vulnerable to chronification, even in individuals who remain physically active.

A second pathway through which physical activity protects against chronic low back pain is at the affective and psychosocial level. Regular exercise is well established to attenuate fear-avoidance behavior, improve self-efficacy, and reduce pain catastrophizing, psychological constructs that are among the strongest independent predictors of pain chronification [25,34,35,36,37]. These effects are not merely motivational; they reflect genuine neurobiological changes in the circuits that regulate threat appraisal, emotional valence of pain, and cognitive control over nociception. The anterior cingulate cortex, prefrontal cortex, and amygdala, key nodes of the affective pain network, are remodeled by repeated exercise-induced experiences of successful movement under conditions of mild discomfort, progressively recalibrating the system away from threat and toward safety [38,39]. NSAIDs, by pharmacologically suppressing the discomfort that normally accompanies exercise, may paradoxically short-circuit this recalibration process. If pain during movement is the very signal through which the nervous system learns that activity is safe and tolerable, then removing that signal removes the opportunity for fear extinction and self-efficacy gains. Under this model, chronic NSAID use does not merely blunt inflammation, it may prevent the experiential learning that exercise is meant to produce at the affective level, maintaining or even entrenching fear-avoidance and catastrophizing rather than resolving them. Future studies examining whether NSAID use moderates exercise-induced changes in fear-avoidance beliefs and self-efficacy scores would directly test this hypothesis.

A third pathway through which NSAIDs may disrupt the protective effects of exercise involves the broader neuroimmune system, and specifically the role of glial cells, particularly microglia, in mediating the transition between acute and chronic pain states. Physical exercise normally elicits a tightly regulated, transient inflammatory response that serves as a biological signal for systemic and central nervous system adaptation [14,40]. Muscle contractions provoke acute increases in pro-inflammatory cytokines such as IL-6, inflammasome activation, and prostaglandin release, which in turn trigger downstream cascades driving tissue repair, mitochondrial biogenesis, and neuroimmune recalibration [41,42]. Critically, this inflammatory drive is not merely peripheral, exercise-induced cytokine and prostaglandin signals cross into the central nervous system, where they engage glial cells and modulate the neuroimmune environment that governs pain sensitivity [41,43]. NSAIDs, by inhibiting COX-enzyme-dependent prostaglandin production and suppressing downstream cytokines including IL-1β and IL-18 [10], and by reducing Toll-like-receptor-induced cytokine secretion by dendritic cells [11], may flatten this signal before it completes its adaptive function, preventing the neuroimmune system from achieving the recalibrated, lower-pain-sensitivity state that exercise is designed to produce.

The central hypothesis is the role of microglia, the brain’s resident immune cells and primary sensors of neuroimmune state [44,45]. Under normal conditions, exercise-induced inflammatory signaling appears to promote adaptive changes in microglial activity toward a more surveillant, homeostatic state associated with neuroprotection and reduced central sensitization [46,47]. Chronic blunting of this signaling by NSAIDs may interfere with these neuroimmune adaptations, although direct evidence that it drives microglia toward a primed, pro-inflammatory phenotype is still limited. Prostaglandin signaling likely contributes to this process in a context-dependent manner, serving not only as an inflammatory mediator but also as a regulator of microglial responses over time [48,49]. The result is a neuroimmune environment that is neither acutely inflamed nor fully resolved, a pathologically stable intermediate state in which the spinal dorsal horn and supraspinal pain centers remain sensitized despite the absence of overt tissue pathology.

This model also helps explain the subgroup heterogeneity observed in our data. Populations with already altered neuroimmune baselines, including older adults, individuals with depression, hypertensive patients, and those with obesity, showed the most pronounced NSAID-by-exercise interactions. Each of these conditions has been independently associated with microglial dysregulation and elevated baseline neuroinflammation [47,50,51]. In such individuals, the exercise-induced neuroimmune effects may be the primary mechanism available to periodically reset glial reactivity and restore descending inhibitory tone. When NSAIDs could plausibly suppress this signal, the system loses its only available recalibration signal, and the protective effect of physical activity is not merely attenuated but attenuated or reversed, a possible interpretation of the signals observed in these subgroups. Trappe & Liu [15] demonstrated that prostaglandins are specifically required for skeletal muscle adaptation to exercise and that COX inhibition alters both acute and long-term training responses, underscoring that this is not a theoretical concern but a pharmacologically demonstrated interference with adaptive biology. Future research using microglial activation markers, central sensitization indices, and inflammatory cytokine profiling in exercise-NSAID interaction studies would directly test whether this diffuse neuroimmune pathway explains the effect modification we observed.

Our findings do not directly measure underlying biological mechanisms but do converge with recent findings and are interpreted in the context of existing literature in other pain conditions. Alves et al. [18] reported that common analgesics, including antidepressants and muscle relaxants, were associated with alterations in endogenous pain-modulatory efficiency in fibromyalgia, while Serrano et al. [19] showed that deficits in the endogenous pain-modulatory system correlate with cognitive impairment and reduced pain inhibition in this population. Together, these studies reinforce the idea that chronic pharmacologic modulation of inflammatory or neuromodulatory systems can reshape endogenous pain control networks. Similarly, meta-analytic evidence indicates that exercise and neuromodulatory interventions such as tDCS enhance cortical excitability and pain inhibition [21,52], supporting the view that physiologic activation, rather than suppression, of these systems is essential for adaptive analgesia.

This study carries meaningful strengths. The use of NHANES 2009–2010 data provides a nationally representative sample of U.S. adults, with survey weighting procedures ensuring that findings generalize to the broader civilian non-institutionalized population rather than a selected clinical cohort. The analytic approach, survey-weighted logistic regression with prespecified interaction terms and subgroup analyses, is methodologically appropriate for the complex NHANES sampling design and was implemented using validated survey procedures. The hypothesis being tested, that NSAID use modifies the association between physical activity and CLBP, is both clinically important and mechanistically grounded, and to our knowledge this interaction has not been previously examined in a nationally representative sample. The breadth of physical activity domains examined (active transport, moderate and vigorous recreational activity, and moderate and vigorous work activity) captures the multidimensional nature of daily movement in a way that single-domain PA measures do not.

As with all cross-sectional analyses, the NHANES design does not permit causal inference, and reverse causation is an inherent possibility: individuals with more severe or treatment-resistant pain may be more likely to use NSAIDs, such that NSAID use partly reflects pain burden. This is a limitation shared by virtually all observational studies of analgesic use in pain populations, and it underscores the value of future longitudinal or interventional designs that can establish directionality. Although the number of tested interactions and exploratory subgroup analyses requires cautious interpretation, the presence of signals across more than one PA and NSAID category supports further investigation. NSAID exposure, while systematically captured through NHANES medication protocols, lacks detail on dosage, duration, and timing relative to physical activity, parameters that are likely mechanistically important and that future pharmacoepidemiology studies should prioritize. Similarly, physical activity was measured through the GPAQ-based PAQ, a validated self-report instrument that, like all self-report measures, carries some degree of measurement error relative to objective accelerometry [53]; future studies incorporating wearable devices alongside medication diaries would substantially strengthen exposure characterization. Although analyses were adjusted for a broad range of sociodemographic and clinical covariates, residual confounding from unmeasured factors, including pain severity, injury history, occupational demands, concurrent medication use, and psychosocial variables, remains possible, as in any observational study of this kind. The large interaction odds ratios in certain subgroups, particularly older adults and individuals with depression, should be interpreted as hypothesis-generating signals warranting dedicated replication in larger stratified samples, rather than precise effect estimates. Taken together, these considerations do not undermine the study’s core findings but rather delineate a well-defined path forward for confirmatory research.

## 5. Conclusions

In a nationally representative sample of U.S. adults, NSAID use was associated with modification of the protective association between physical activity and chronic low back pain. These findings should be interpreted as hypothesis-generating and do not establish that NSAIDs causally interfere with the protective effects of physical activity. Rather, they suggest that NSAIDs may be correlated with multiple pathways through which exercise confers protection: blunting the afferent sensory signals that drive motor cortical plasticity and descending inhibitory tone, modifying the experiential learning through which exercise recalibrates fear-avoidance and catastrophizing, and suppressing the transient neuroimmune pulse through which physical activity resets glial reactivity and restores central pain regulation. However, these pathways were not directly tested in the present study. Given the observational and cross-sectional design, reverse causation and residual confounding remain important considerations. Individuals with more severe or persistent pain may be more likely to use NSAIDs, and medication use may therefore reflect underlying pain burden rather than a causal modifier of exercise-related benefit. Future longitudinal and randomized trials are needed to evaluate causality, define the dose and timing thresholds at which NSAID use begins to attenuate exercise benefit, and identify anti-inflammatory strategies that preserve rather than suppress the adaptive biology through which movement heals.

## Figures and Tables

**Table 1:** Characteristics of U.S. adults with and without chronic low back pain, NHANES 2009–2010.

Variable	No CLBP (n)	CLBP (n)	Total (n, Weighted % ± SE)
Age ≥ 60 years	26.0	182.0	208 (16.5 ± 1.1)
Male	99.0	363.0	462 (48.3 ± 1.7)
Female	91.0	435.0	526 (51.7 ± 1.7)
Current smoker	93.0	486.0	579 (55.8 ± 2.2)
Depression (PHQ-9)	25.0	174.0	199 (19.7 ± 1.3)
Obesity (BMI ≥ 30)	80.0	370.0	450 (43.5 ± 1.7)
High blood pressure	58.0	325.0	383 (34.5 ± 1.9)
Married/partnered	115.0	465.0	580 (62.7 ± 2.8)
Any NSAID use	146.0	694.0	840 (85.5 ± 1.9)
Ibuprofen use	129.0	631.0	760 (78.8 ± 2.3)
Naproxen use	81.0	424.0	505 (53.1 ± 2.5)
COX-2 inhibitor use	12.0	156.0	168 (17.6 ± 2.3)
Aspirin use	52.0	261.0	313 (33.0 ± 1.8)
Vigorous recreational	40.0	111.0	151 (17.9 ± 1.9)
activity			
Moderate recreational	113.0	413.0	526 (58.2 ± 1.7)
activity			
Active transport ≥ 10	64.0	169.0	233 (21.4 ± 1.7)
min/day			
Moderate work activity	93.0	355.0	448 (48.3 ± 1.6)
Vigorous work activity	41.0	192.0	233 (26.3 ± 1.6)
Subgroup: Non-smoker	97.0	312.0	409 (44.2 ± 2.2)
Subgroup: Depression: No	147.0	543.0	690 (80.3 ± 1.3)
Subgroup: Not partnered	75.0	333.0	408 (37.3 ± 2.8)
Subgroup: Age < 60 years	164.0	616.0	780 (83.5 ± 1.1)
Subgroup: Non-obese (BMI < 30)	105.0	413.0	518 (56.5 ± 1.7)

Values are unweighted counts (n) by chronic low-back-pain status. The Total column presents survey-weighted prevalence estimates (% ± SE) for the overall U.S. adult population, calculated using NHANES interview weights (WTINT2YR) and accounting for the complex sampling design (strata = SDMVSTRA, PSU = SDMVPSU; Taylor linearization).

**Figure A1:**
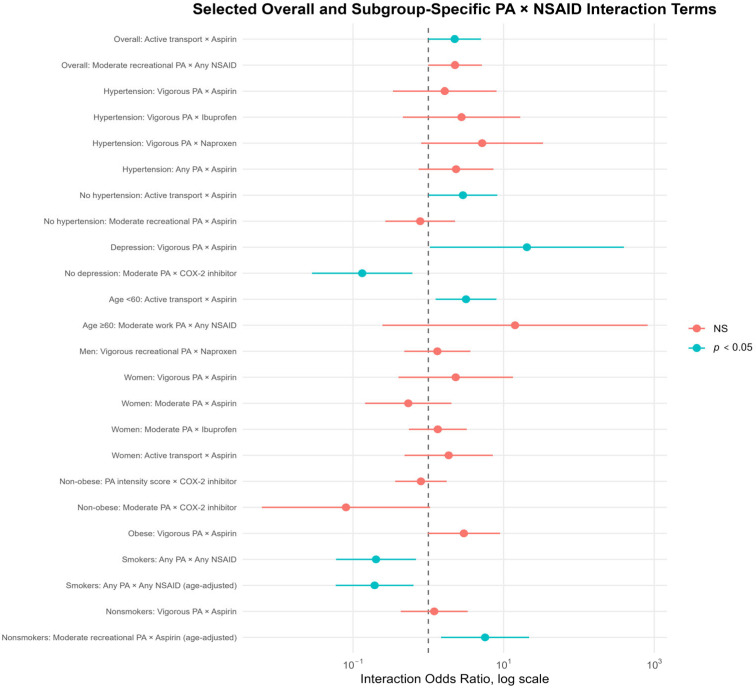
Forest plot of selected overall and subgroup-specific PA × NSAID interaction terms for chronic low back pain (log-scale odds ratios). Points represent interaction odds ratios and horizontal lines represent 95% confidence intervals. Point color indicates statistical significance (*p* < 0.05 vs. NS). The dashed vertical line indicates the null interaction value, OR = 1.

**Figure A2:**
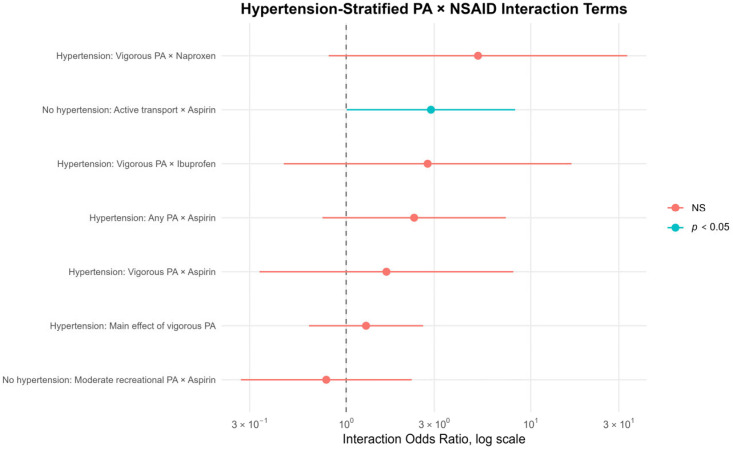
Hypertension-stratified forest plot of selected PA × NSAID interaction terms for chronic low back pain. Points represent interaction odds ratios and horizontal lines represent 95% confidence intervals. Point color indicates statistical significance (*p* < 0.05 vs. NS). The dashed vertical line indicates the null interaction value, OR = 1.

**Figure A3:**
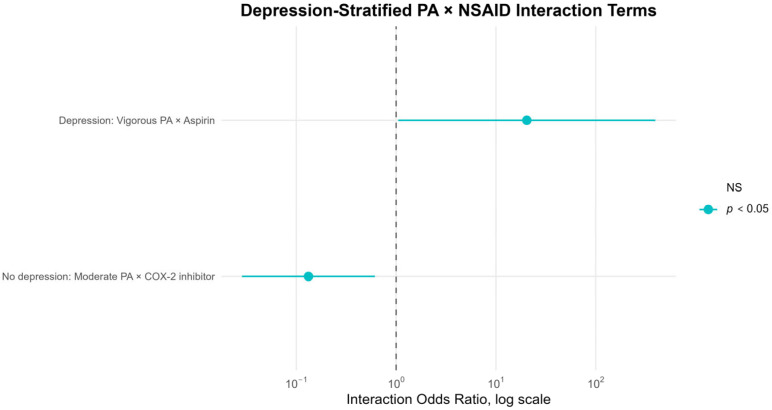
Depression-stratified forest plot of selected PA × NSAID interaction terms for chronic low back pain. Points represent interaction odds ratios and horizontal lines represent 95% confidence intervals. Point color indicates statistical significance (*p* < 0.05). The dashed vertical line indicates the null interaction value, OR = 1.

## Data Availability

NHANES 2009–2010 datasets are publicly available at: https://wwwn.cdc.gov/nchs/nhanes/continuousnhanes/Default.aspx (accessed on 18 May 2026).

## Abbreviations

The following abbreviations are used in this manuscript:

BMI	body mass index
CDC	Centers for Disease Control and Prevention
CI	confidence interval
CLBP	chronic low back pain
COX-2	cyclooxygenase-2
GPAQ	Global Physical Activity Questionnaire
NHANES	National Health and Nutrition Examination Survey
NSAID	non-steroidal anti-inflammatory drug
OR	odds ratio
PA	physical activity
PAQ	Physical Activity Questionnaire
PHQ-9	Patient Health Questionnaire-9
PSU	primary sampling unit
SE	standard error

## Appendix A

Online Resource Table A1. Unweighted baseline characteristics of U.S. adults with and without chronic low back pain, NHANES 2009–2010: Values represent unweighted sample counts and percentages by chronic low back pain status. Unlike weighted analyses (Taylor linearization method), these figures describe raw sample composition and are not adjusted for the NHANES complex survey design (WTINT2YR, SDMVSTRA, SDMVPSU).

**Table A1:** Unweighted baseline characteristics of U.S. adults with and without chronic low back pain, NHANES 2009–2010.

Variable	No Chronic LBP (N = 190)	Chronic LBP (N = 798)	Total (N = 988)
Any NSAID Use: No	44 (23.2%)	104 (13.0%)	148 (15.0%)
Any NSAID Use: Yes	146 (76.8%)	694 (87.0%)	840 (85.0%)
Aspirin Use: No	138 (72.6%)	535 (67.0%)	673 (68.1%)
Aspirin Use: Yes	52 (27.4%)	261 (32.7%)	313 (31.7%)
Ibuprofen Use: No	61 (32.1%)	167 (20.9%)	228 (23.1%)
Ibuprofen Use: Yes	129 (67.9%)	631 (79.1%)	760 (76.9%)
Naproxen Use: No	109 (57.4%)	370 (46.4%)	479 (48.5%)
Naproxen Use: Yes	81 (42.6%)	424 (53.1%)	505 (51.1%)
Cox-2 Inhibitor Use: No	178 (93.7%)	631 (79.1%)	809 (81.9%)
Cox-2 Inhibitor Use: Yes	12 (6.3%)	156 (19.5%)	168 (17.0%)
Indomethacin Use: No	188 (98.9%)	762 (95.5%)	950 (96.2%)
Indomethacin Use: Yes	2 (1.1%)	36 (4.5%)	38 (3.8%)
Physical Activity			
Walk/Bike ≥ 10 min: No	126 (66.3%)	629 (78.8%)	755 (76.4%)
Walk/Bike ≥ 10 min: Yes	64 (33.7%)	169 (21.2%)	233 (23.6%)
Moderate Recreational PA: No	117 (61.6%)	496 (62.2%)	613 (62.1%)
Moderate Recreational PA: Yes	73 (38.4%)	302 (37.8%)	375 (38.0%)
Vigorous Recreational PA: No	150 (78.9%)	687 (86.1%)	837 (84.7%)
Vigorous Recreational PA: Yes	40 (21.1%)	111 (13.9%)	151 (15.3%)
Moderate Work PA: No	138 (72.6%)	635 (79.6%)	773 (78.3%)
Moderate Work PA: Yes	52 (27.4%)	163 (20.4%)	215 (21.8%)
Vigorous Work PA: No	149 (78.4%)	606 (75.9%)	755 (76.4%)
Vigorous Work PA: Yes	41 (21.6%)	192 (24.1%)	233 (23.6%)
Subgroups			
Sex: Male	99 (52.1%)	363 (45.5%)	462 (46.8%)
Sex: Female	91 (47.9%)	435 (54.5%)	526 (53.2%)
Smoking Status: Non-smoker	97 (51.1%)	312 (39.1%)	409 (41.4%)
Smoking Status: Smoker	93 (48.9%)	486 (60.9%)	579 (58.6%)
Depression Status: No	147 (77.4%)	543 (68.0%)	690 (69.8%)
Depression Status: Yes	25 (13.2%)	174 (21.8%)	199 (20.1%)
Married/Partnered: No	75 (39.5%)	333 (41.7%)	408 (41.3%)
Married/Partnered: Yes	115 (60.5%)	465 (58.3%)	580 (58.7%)
High Blood Pressure: No	132 (69.5%)	473 (59.3%)	605 (61.2%)
High Blood Pressure: Yes	58 (30.5%)	325 (40.7%)	383 (38.8%)
Age Group: <60	164 (86.3%)	616 (77.2%)	780 (78.9%)
Age Group: ≥60	26 (13.7%)	182 (22.8%)	208 (21.1%)
Obesity: Non-obese	105 (20.27%)	413 (79.3%)	518 (52.43%)
Obesity: Obese	80 (17.78%)	370 (82.2%)	450 (45.55%)

Subgroup analyses are displayed in this section. These analyses were exploratory and should be interpreted cautiously because several estimates had wide confidence intervals or were sensitive to model specification. We highlight the most pronounced findings.

## Appendix B

Hypertension: the modifying effect of NSAIDs on the PA–CLBP relationship varied by blood pressure status, but several previously described large effects were not supported by the matched Excel output. Among individuals without hypertension history, active transport × aspirin was significant (OR = 2.88, 95% CI 1.01–8.23; *p* = 0.049), but the hypertension group is suggestive of inconsistent attenuation.

Depression: among participants with depression, vigorous PA × aspirin produced a large interaction estimate (OR = 20.38, 95% CI 1.05–395.42; *p* = 0.047). Because the confidence interval was extremely wide, this finding should be interpreted as a large exploratory signal rather than a precise estimate of risk. Among participants without depression, moderate PA × COX-2 inhibitor use showed a protective interaction (OR = 0.13, 95% CI 0.03–0.61; *p* = 0.014). These subgroup differences are hypothesis-generating and may reflect differences in baseline neuroimmune state, medication patterns, or sparse-cell instability.

Age: The effect modification by NSAIDs differed by age. In younger adults (<60 years), active transport × aspirin was significant (OR = 3.17, 95% CI 1.25–8.02; *p* = 0.019). In older adults (≥60 years), moderate work-related PA × any NSAID use produced a large but highly imprecise estimate that was not statistically significant (OR = 14.17, 95% CI 0.25–817.07; *p* = 0.176). Therefore, the age-stratified findings support cautious interpretation of possible attenuation in younger adults and unstable estimates in older adults, rather than a definitive extreme interaction. Sex: Significant sex-specific reversals were not found.

Obesity: In non-obese adults (BMI < 30), the PA-number × COX-2 interaction was not significant (OR = 0.80, 95% CI 0.36–1.75; *p* = 0.545). A sensitivity model for moderate PA × COX-2 showed a protective direction but remained borderline (OR = 0.08, 95% CI 0.006–1.05; *p* = 0.054). Among obese adults (BMI ≥ 30), vigorous PA × aspirin showed a harmful direction but was borderline after rounding (OR = 2.97, 95% CI 0.98–8.97; *p* = 0.053). These findings should be framed as suggestive and imprecise rather than definitive evidence of obesity-specific reversal.

Smoking: Non-smokers exhibited a strong reversal effect for vigorous exercise with NSAIDs. Smoking-stratified models showed divergent patterns. In smokers, any PA × any NSAID use showed a protective interaction when adjusted for age (OR = 0.20, 95% CI 0.06–0.69; *p* = 0.015), with a similar result using age group adjustment (OR = 0.19, 95% CI 0.06–0.64; *p* = 0.012). In non-smokers, vigorous PA × aspirin was not significant (OR = 1.20, 95% CI 0.43–3.34; *p* = 0.707).
